# Examining Impacts of Allergic Diseases on Psychological Problems and Tobacco Use in Korean Adolescents: The 2008–2011 Korean National Health and Nutrition Examination Survey

**DOI:** 10.1371/journal.pone.0125172

**Published:** 2015-04-21

**Authors:** Yoon Hong Chun, Kyungdo Han, Yong-Gyu Park, Jong-seo Yoon, Hyun Hee Kim, Jin Tack Kim, Dae Chul Jeong

**Affiliations:** 1 Department of Pediatrics, Incheon St. Mary’s Hospital, College of Medicine, The Catholic University of Korea, Seoul, Republic of Korea; 2 Department of Biostatistics, The Catholic University of Korea, Seoul, Republic of Korea; 3 Department of Pediatrics, Seoul St. Mary’s Hospital, College of Medicine, The Catholic University of Korea, Seoul, Republic of Korea; 4 Department of Pediatrics, Bucheon St. Mary’s Hospital, College of Medicine, The Catholic University of Korea, Seoul, Republic of Korea; 5 Department of Pediatrics, Uijeongbu St. Mary’s Hospital, College of Medicine, The Catholic University of Korea, Seoul, Republic of Korea; University of Alabama at Birmingham, UNITED STATES

## Abstract

**Purpose:**

Asthma during adolescence can induce social, psychological, and behavioral problems. We examined the impact of asthma and other allergic diseases on psychological symptoms and health risk behaviors among South Korean adolescents.

**Methods:**

In this population-based cross-sectional study, 3192 adolescents (10–18 years of age) participating in the 2008–2011 Korean National Health and Nutrition Examination Survey were enrolled. Psychological problems associated with clinically diagnosed asthma, allergic rhinitis, and atopic dermatitis were assessed using questionnaires and surveys. Data was analyzed using logistic regression to determine the association of depression with allergic disease while controlling for age, sex, body mass index, smoking experience, and alcohol use.

**Results:**

Asthma and atopic dermatitis were associated with a higher prevalence of depression (17.2% and 13%, respectively). After adjusting for the covariates, asthma patients were approximately two times as likely to have depression as non-allergic participants (odds ratio, 1.81; 95% confidence interval, 1.22–2.68). Psychosocial stress significantly increased in the following order: no allergy, any allergy without asthma, asthma only, and asthma with any allergy (p for linear trend = 0.01). The asthma without other allergies group showed the highest prevalence of cigarette smoking (p = 0.007).

**Conclusions:**

In this study, asthma with or without other allergies was significantly related to increases in depression, psychosocial stress, and smoking experience. Thus, care should be taken to adjust treatment to account for the psychological symptoms and health risk behaviors common among asthmatic adolescents.

## Introduction

The Asia-Pacific region houses approximately 60% of the world’s population. Although the prevalence of asthma has been reduced in the West, some countries in Asia, such as China, Indonesia, Taiwan, and South Korea, have shown a rapid growth in prevalence rates of the disease as a result of increasing industrialization and the widespread adoption of Western lifestyles [1,2]. Adolescents in these countries have shown particular increases in asthma symptom prevalence in recent years, while rates among their Western counterparts remain relatively stable [1].

In order to combat this comparatively new healthcare threat, Korea, the most Westernized country within Asia, has adopted a range of medication and evidence-based treatment guidelines from Western countries. While the introduction of asthma care has successfully reduced asthma severity, it is currently unable to effectively deal with the subsequent reductions in quality of life and the emotional impact of the disease [2]. The psychological and social impact of allergic disease among adolescents has been continuously investigated in Western countries [3–7], with results showing that the prevalence of comorbid anxiety and depressive disorders in adolescents with asthma is nearly twice as high as that in healthy youths [4]. Other conditions, including allergic sensitization [5], atopic dermatitis [6], and allergic rhinitis [7], have also been shown to be associated with depression in both children and adults.

Mental health comorbidity in patients with asthma and other allergic diseases leads to deterioration in disease control and quality of life [3,8,9]. Comorbid depressive symptoms and psychological stress in adolescents with allergic disease have been linked to a range of adverse outcomes, including social and educational impairments, physical and mental health problems during adulthood, and heightened risk for suicide [10,11]. Given the wide-ranging implications, the relationship between asthma and depression in adolescents needs to be better understood in order to be able to successfully address the adverse effects of comorbidity among asthmatic adolescents. To date, however, most studies conducted have only examined this association within Western populations and, to our knowledge, no such study has been undertaken to examine the association between asthma and mental health problems in a large sample of South Korean adolescents.

The objective of this study was therefore to use nationally representative data in order to investigate the impact of asthma and other allergic diseases on psychological symptoms and health risk behaviors among adolescents in South Korea.

## Methods

### Subjects

This cross sectional study included a representative sample of the data gathered in the Korean National Health and Nutrition Examination Survey (KNHANES) conducted every year from 2008 to 2011 by the Division of Chronic Disease Surveillance under the Korean Centers for Disease Control and Prevention. The sampling units were registered households from the 2010 National Census Registry selected through a stratified, multistage probability sampling based on geographic area, sex, and age. The sample was representative of the nationwide non-institutionalized civilian Korean population.

Of 37,753 individuals who participated in four cross-sectional surveys, 3192 adolescents aged 10–18 years were enrolled into the study. The Institutional Review Board/Ethics Committee of the Catholic University of Korea approved the study protocol, which was in accordance with the Declaration of Helsinki. The data used are publicly available from the Korean Centers for Disease Control and Prevention [12].

### Procedure

The KNHANES consisted of the three main components of health interview, health examination and nutrition survey. Information was collected regarding demographic details, residential district (i.e., urban or rural), socioeconomic status, cigarette use, alcohol use, sleep duration, and exercise frequency. A responsible adult (usually a parent) provided proxy responses for children included in the survey.

Anthropometric measurements were collected from subjects by a specially trained examiner. Waist circumference was measured to the nearest 0.1 cm in a horizontal plane at the level of the midpoint between the iliac crest and the costal margin at the end of a normal expiration. Body mass index (BMI) was calculated as the individual’s weight in kilograms divided by the square of the individual’s height in meters.

Regular exercise was defined as >30 minutes of physical activity for more than one day a week during the last week. Total calorie intake was recorded with a 24-h dietary recall questionnaire administered by a trained dietician. Cigarette and alcohol use was defined as ever having smoked or drank alcohol.

### Study Variables

Subjects that answered “yes” to the following question were categorized as having asthma (i.e., allergic rhinitis and atopic dermatitis): “Has a doctor or health professional ever told you that you have asthma?” Subjects that answered “yes” to the following question were categorized as depressed: “During the past year, has your daily life been burdened by feelings of hopelessness or dejection for more than two continuous weeks?” In addition, participants were also asked to respond “yes” or “no” to the following mental health questions: “Have you ever thought about suicide?”; “Have you engaged in a suicide attempt during the past year?”; and “Have you had psychiatric counseling during the past year?” Psychological stress was evaluated from responses indicative of cognitive complaints collected with a questionnaire using a four-point Likert scale: “excessive,” “much,” “frequent” and “rarely.” Additionally, in order to analyze the associations of depression, psychological stress, and health risk behavior with allergic disease, we categorized respondents into four asthma groups: “asthma with any allergy,” “asthma only,” “any allergy without asthma,” and “no allergy or asthma.”

### Statistical Analyses

Data are expressed as percentages and standard error (categorical) or the mean ± standard error (continuous). Multivariable adjusted logistic regression analysis was conducted to calculate odds ratios (ORs) and 95% confidence intervals (CIs) for the association of asthma with stress, depression, suicidal ideation, and psychiatric counseling. Age, sex, BMI, and smoking and alcohol experience were covariates used to calculate adjusted ORs.

Weighted analyses were performed given that the KNHANES included weights to compensate for the complex sampling design and to allow for nationally representative prevalence estimates of the Korean population. All statistical analyses were conducted using the Statistical Analysis System (SAS version 9.2; SAS Institute, Cary, NC, USA). A p-value of < 0.05 was considered statistically significant.

## Results

### Baseline Characteristics

As Korea is a single-race nation, all of the subjects were Asian. A total of 293 participants were identified as having asthma and included in the asthma group; 2899 were identified as not having asthma and were included in the non-asthma group. There were no significant differences between the two groups in terms of age, height, weight, BMI, waist circumference, blood pressure, daily calorie intake, area of residence, socioeconomic status, alcohol experience, sleep duration, or exercise frequency (Table 1). However, those in the asthma group were significantly more likely to have atopic dermatitis (16.1% versus 9.6%; p = 0.002) and allergic rhinitis (47.4% versus 31.3%; p < 0.001). The asthma group reported having significantly more smoking experience compared to the non-asthma group (27.9% versus 17.8%; p < 0.001). Further, the asthma group included significantly more males (p = 0.04).

**Table 1:** Sociodemographic and clinical characteristics for Korean adolescents with and without asthma.

	Asthma		p
	Yes (*n* = 293)^a^	No (*n* = 2899)^a^	
Age	15.2 ± 0.1	15 ± 0	0.11
Height	165.8 ± 0.6	164.7 ± 0.2	0.10
Weight	59 ± 0.9	57.3 ± 0.3	0.07
BMI^b^	21.3 ± 0.3	21 ± 0.1	0.18
Waist circumference	71.7 ± 0.7	70.7 ± 0.2	0.15
Systolic BP^b^	107.5 ± 0.7	107.4 ± 0.3	0.90
Diastolic BP	68.5 ± 0.6	67.9 ± 0.2	0.28
Calorie intake/day	2204.1 ± 66.5	2104.1 ± 23.1	0.15
Male	60.1 (3.1)	53.1 (1.1)	0.04
Rural residence	15.8 (2.7)	17.3 (2)	0.59
Income, lowest quartile	18.7 (3)	15 (1.1)	0.19
Ever smoked	27.9 (3.2)	17.8 (0.9)	<0.001
Ever drank alcohol	33.7 (3.5)	27.7 (1.1)	0.08
Sleep duration			0.58
<6 h/d	13 (2.2)	11.2 (0.7)	
6 to 8 h/d	68.8 (3.3)	71.8 (1)	
>8 h/d	18.2 (2.5)	17.1 (0.9)	
Regular exercise	34.7 (3.3)	29.6 (1)	0.13
AD^b^	16.1 (2.4)	9.6 (0.7)	0.002
AR^b^	47.4 (3.7)	31.3 (1.2)	<0.001
AD or AR	47.6 (3.5)	40.0 (1.3)	0.007

^a^Data are presented as means ± SE or percentages (SE).

^b^BMI, body mass index; BP, blood pressure; AD, atopic dermatitis; AR, allergic rhinitis.

### Psychological Problems Associated with Allergic Diseases

The prevalence of depression was significantly higher in adolescents with asthma compared to those without (17.2% versus 9.8%; p < 0.001). However, there were no significant differences between the two groups in terms of stress level, psychiatric counseling, suicidal ideation, and suicidal attempts. Depression was significantly more prevalent among those with atopic dermatitis than among those without it (13% versus 8.6%; p = 0.003), as were suicide attempts (1.4% versus 0.5%; p = 0.03). The prevalence of psychological problems did not differ between subjects with and without non-allergic rhinitis (Table 2).

**Table 2:** Prevalence of psychological problems in Korean adolescents with allergic diseases.

	Asthma			Allergic rhinitis			Atopic dermatitis		
	Yes^a^	No^a^	p	Yes^a^	No^a^	p	Yes^a^	No^a^	p
**Stress**			0.12			0.39			0.08
Excessive	6.3 (1.8)	1.6 (0.8)		4.3 (1.1)	4 (0.5)		5.3 (0.9)	3 (0.4)	
Much	26.8 (2.9)	23.6 (1)		27.9 (2.7)	23.5 (1)		24 (1.7)	23.4 (1.2)	
Frequent	50.7 (3.4)	57.6 (1.1)		54.5 (3.3)	57.3 (1.1)		56.1 (2)	58 (1.3)	
Rarely	16.2 (2.7)	15 (0.8)		13.2 (2.2)	15.3 (0.8)		14.7 (1.6)	15.6 (1)	
**Depression**	17.2 (2.7)	9.8 (0.7)	<0.001	9.9 (1.7)	10.5 (0.7)	0.74	13 (1.5)	8.6 (0.8)	0.003
**Psychiatric counseling**	3.7 (1)	2.9 (0.4)	0.45	4.5 (1.5)	2.8 (0.4)	0.18	4 (0.8)	2.7 (0.5)	0.12
**Suicidal ideation**	14.3 (2.3)	13 (0.8)	0.59	14.2 (2.1)	13 (0.8)	0.57	14.4 (1.4)	12.2 (0.9)	0.17
**Suicidal attempt**	1.6 (0.8)	0.7 (0.2)	0.09	0.8 (0.7)	0.8 (0.2)	0.91	1.4 (0.6)	0.5 (0.2)	0.03

^a^Data are presented as percentages (SE).

### Associations between Asthma and Depression

**Fig 1:**
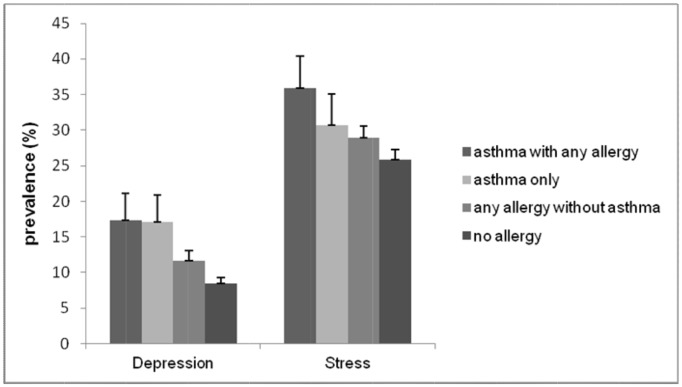
The prevalence of depression and psychological stress among adolescents by allergic disease status. The error bars represent the upper 95% confidence intervals. The group that includes asthma had a significantly higher prevalence of depression and stress compared to the group that did not include asthma (depression, p for linear trend < 0.001; stress, p for linear trend = 0.01).

**Fig 2:**
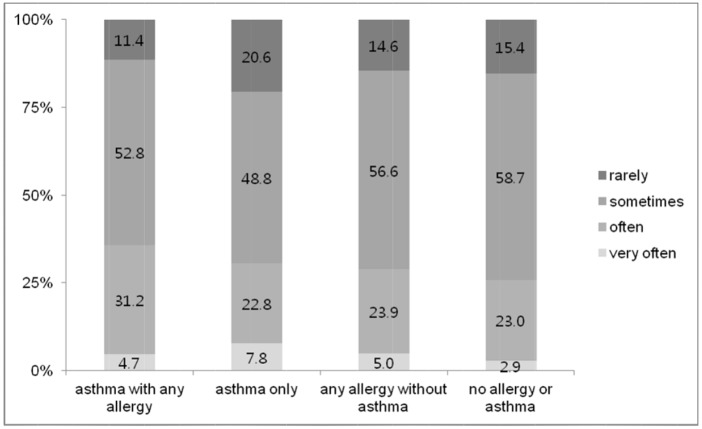
The distribution of psychological stress levels among adolescents by allergic disease status. The group that includes asthma were stressed more frequently than the group that did not include asthma (p for linear trend = 0.03).

The association between asthma and psychological problems was examined using logistic regression analysis (Table 3). Our results showed that the risk of depression was higher in the asthma group when age, sex, BMI, smoking, and alcohol use were included in a multivariate model (adjusted OR = 1.81, CI 95% = 1.22–2.68), whereas stress level, suicidal ideation, and psychiatric counseling were not. We also analyzed the association of depression and stress level with allergic disease by classifying allergic status into four groups: “asthma with any allergy,” “asthma only,” “any allergy without asthma,” and “no allergy or asthma.” The group that included asthma had a significantly higher prevalence of depression (p for linear trend < 0.001) and stress (p for linear trend = 0.01) compared to the group that did not include asthma (Fig 1). In the case of stress, the group that included asthma were significantly stressed more frequently than the group that did not include asthma (p for linear trend = 0.03; Fig 2).

**Table 3:** Odds ratio (95% CI) of multivariate analysis analyzing the association between asthma and psychological problems.

	Model 1^a^	Model 2^b^	Model 3^c^	Model 4^d^
Stress	1.312 (0.98,1.75)	1.334 (1.00,1.79)	1.325 (0.98,1.79)	1.282 (0.94,1.74)
Depression	1.91 (1.30,2.82)	1.918 (1.30,2.84)	1.921 (1.30,2.85)	1.81 (1.22,2.68)
Suicidal ideation	1.111 (0.76,1.63)	1.166 (0.79,1.72)	1.159 (0.78,1.71)	1.087 (0.73,1.61)
Psychiatric counseling	1.271 (0.68,2.38)	1.300 (0.69,2.44)	1.283 (0.69,2.39)	1.184 (0.64,2.19)

^a^Model 1: unadjusted.

^b^Model 2: adjusted for demographic date (age and sex).

^c^Model 3: model 2 + adjusted for obesity (body mass index).

^d^Model 4: model 3 + adjusted for health risk behaviors (smoking and alcohol use).

### Associations between Allergic Disease and Health Risk Behaviors

The group that included asthma had significantly higher rates of cigarette use than the group that did not include asthma (p = 0.007). Specifically, the “asthma only” group showed the highest rate of smoking among the four groups. In addition, the group that included asthma was more likely to have alcohol use experience than the group that did not include asthma. However, there were no significant differences among the four groups (Table 4).

**Table 4:** Association between allergic disease, cigarette smoking and alcohol use in Korean adolescents.

	Group 1^a^	Group 2^b^	Group 3^c^	Group 4^d^	p
Ever smoked	25.1(4.4)	30.4(4.7)	17.5(1.6)	17.8(1.2)	0.007
Ever drank alcohol	29.1(4.7)	37.8(4.9)	26.9(1.8)	27.7(1.5)	0.15

^a-d^Data is presented as percentages (SE).

^a^Group 1: asthma with any allergy

^b^Group 2: asthma only

^c^Group 3: any allergy without asthma

^d^Group 4: no allergy

## Discussion

In the current representative study of the South Korean population, comorbid depression was significantly more prevalent among adolescents with asthma and atopic dermatitis than among adolescents without these conditions. This result is consistent with previous research conducted in Western populations [3–5]. The association between depression and asthma, however, was only confirmed when age, sex, BMI, and smoking and alcohol experience confounds were included in the multivariate model. Even when we compared all four classifications of allergic disease, only the asthma groups with or without other allergic disease showed a significantly higher prevalence of depression and psychological stress than the groups without asthma. Likewise, with regard to health risk behaviors, the groups that included asthma also reported significantly more smoking experience.

The existence of a relationship between allergic disease and depression has been suggested for many years. Hurwitz et al. recruited 6836 patients comorbid for allergy and depression and reported that subjects with a history of any allergy (i.e., asthma or allergic rhinitis) were more likely to be diagnosed with major depression [13]. When assessing a cohort of elderly asthma patients, Kraustkopf et al. concluded that depressive symptoms were associated with poorer asthma control and lower quality of life, as well as with lower rates of adherence to controller medications [14]. Furthermore, Juniper et al revealed that adolescents with allergic rhinitis demonstrated greater attention difficulty, psychomotor slowing, poor sleep, daytime tiredness, irritability, anxiety, and depression [15].

One potential mechanism underlying comorbid asthma and depression is exposure to adversity. Children with asthma experience greater emotional burden or frustration because of their limited physical activity and body dissatisfaction [16]. Asthma interferes with normal development and growth by limiting participation in physical activities and social activities, which can negatively affect childhood relationships and self-esteem [17]. Our study supports this observation, showing that adolescents with asthma, regardless of whether they had any other allergic disease, experienced more psychological stress than did adolescents without asthma. This higher level of stress can be the consequence of poor adaptation to the developmental challenges associated with maturation.

In addition, environmental factors such as air pollution are implicated in the pathogenesis of depression in asthma. Recent research provides evidence that prolonged exposure to particulate matter air pollutants results in depressive-like affective responses as well as pulmonary inflammation through upregulation of pro-inflammatory cytokine expression [18]. Furthermore, Individuals who are vulnerable to major depressive disorder and asthma are influenced by the dysregulation of the autonomic nervous and immune systems, which are sensitive to the stress of chronic illness [19]. However, our analysis, based on genetic homogeneity of a homogenous society, did not show significant differences between asthma patients and normal controls in anthropometric measurements, socioeconomic state, area of residence and lifestyle.

The high rate of depression in adolescents with asthma found in this study is worrisome because depressive disorder can worsen asthmatic symptoms, making them even more difficult to control. A study of inner city asthma patients showed that patients with a psychiatric diagnosis were significantly associated with frequent emergency room visits for asthma treatment and greater use of short acting β2-agonists [20]. Other large population-based studies of adolescents with asthma showed that depression is associated with significantly more asthma symptom days and burden [21]. Furthermore, depression in individuals with asthma is a risk factor for lower adherence to controller medications [14].

Our adjusted analysis revealed that asthma patients had significantly higher rates of cigarette smoking than did not only those without asthma, but also those with other allergic diseases. These results extend the findings of a prior study showing that adolescents and young adults with asthma had higher rates of having tried cigarette smoking compared to those without asthma [22]. Smoking may cause asthma medication failure and contribute to a greater prevalence of asthmatic symptoms [4]. Therefore, considering allergic patients’ quality of life and financial cost to the health system, more care should be invested in those who smoke.

Although our study used a nationally representative population-based sample with ostensibly uniform genetic and environmental influences, it has several limitations that should be taken into consideration. First, the definition of depression used in this study did not correspond to the clinical diagnostic criterion of depression. Nonetheless, the characteristics of our depression items have been shown to be consistent with other research that used questionnaires to assess depression [23,24]. Second, we could not compare atopic asthma and non-atopic asthma due to the lack of specific immunoglobulin E (IgE) “skin prick test” data. However, we categorized subjects into four groups that reflected patients’ other allergic disease status. Asthma patients with a positive history of either atopic dermatitis or allergic rhinitis could be regarded as having allergic asthma [9]. Third, because the original data did not include duration of disease or a detailed description of medication use, we could not determine the relationship between asthma severity and depression. However, prior studies that report the impact of depression in youths with asthma have shown that asthma severity is not associated with depression or anxiety [4,21,25]. Finally, we used cross-sectional data that was not designed to exclusively investigate the psychological impact of the disease. This prevents any causal interpretation and the use of additional key variables. Further longitudinal follow-up studies of adolescents are needed to examine the temporal relationship between allergic disease and psychological problems.

Among all of the allergic diseases assessed in adolescents, only asthma showed a significant association with depression, psychological stress, and smoking. Depressive symptoms of young patients may negatively affect asthmatic outcomes and medication adherence [14]. Psychological stress, as well as experience with smoking cigarettes, can cause asthmatic symptom aggravation through an enhancement of allergic inflammatory responses [26]. Furthermore, the psychological effects and health risk behaviors that begin in adolescence can cause functional impairment in adulthood, as well as result in an increased national socioeconomic burden. Thus, clinicians who work with asthmatic patients should be aware of the high level of comorbidity between depression and asthma and make themselves aware of what this relationship means in practice in order to effectively improve treatment of asthmatic patients.
